# Aero-Ga_2_O_3_ Nanomaterial Electromagnetically Transparent from Microwaves to Terahertz for Internet of Things Applications

**DOI:** 10.3390/nano10061047

**Published:** 2020-05-29

**Authors:** Tudor Braniste, Mircea Dragoman, Sergey Zhukov, Martino Aldrigo, Vladimir Ciobanu, Sergiu Iordanescu, Liudmila Alyabyeva, Francesco Fumagalli, Giacomo Ceccone, Simion Raevschi, Fabian Schütt, Rainer Adelung, Pascal Colpo, Boris Gorshunov, Ion Tiginyanu

**Affiliations:** 1 National Center for Materials Study and Testing, Technical University of Moldova, Stefan cel Mare av. 168, 2004 Chisinau, Moldova; vladimir.ciobanu@cnstm.utm.md; 2 National Institute for Research and Development in Microtechnologies (IMT Bucharest), Erou Iancu Nicolae Street 126A, 077190 Voluntari, Romania; mircea.dragoman@imt.ro (M.D.); martino.aldrigo@gmail.com (M.A.); sergiu.iordanescu@imt.ro (S.I.); 3 Laboratory of Terahertz Spectroscopy, Center for Photonics and 2D Materials, Moscow Institute of Physics and Technology (State University), 9 Institutskiy per., 141701 Dolgoprudny, Russia; zs1978@mail.ru (S.Z.); aliabeva.ln@mipt.ru (L.A.); gorshunov.bp@mipt.ru (B.G.); 4 European Commission, Joint Research Centre (JRC), Via E. Fermi, 2749, 21027 Ispra, Italy; francesco-sirio.fumagalli@ec.europa.eu (F.F.); giacomo.ceccone@ec.europa.eu (G.C.); pascal.colpo@ec.europa.eu (P.C.); 5 Department of Physics and Engineering, State University of Moldova, Alexei Mateevici str. 60, 2009 Chisinau, Moldova; raevskis@mail.ru; 6 Institute for Materials Science, Kiel University, Kaiserstr. 2, D-24143 Kiel, Germany; fas@tf.uni-kiel.de (F.S.); ra@tf.uni-kiel.de (R.A.); 7 Academy of Sciences of Moldova, Stefan cel Mare av. 1, MD-2001 Chisinau, Moldova

**Keywords:** aero-Ga_2_O_3_, ultra-porous nanomaterial, extremely low reflectivity, electromagnetically transparent nanomaterial, X-band and terahertz frequencies

## Abstract

In this paper, fabrication of a new material is reported, the so-called Aero-Ga_2_O_3_ or Aerogallox, which represents an ultra-porous and ultra-lightweight three-dimensional architecture made from interconnected microtubes of gallium oxide with nanometer thin walls. The material is fabricated using epitaxial growth of an ultrathin layer of gallium nitride on zinc oxide microtetrapods followed by decomposition of sacrificial ZnO and oxidation of GaN which according to the results of X-ray diffraction (XRD) and X-ray photoemission spectroscopy (XPS) characterizations, is transformed gradually in *β*-Ga_2_O_3_ with almost stoichiometric composition. The investigations show that the developed ultra-porous Aerogallox exhibits extremely low reflectivity and high transmissivity in an ultrabroadband electromagnetic spectrum ranging from X-band (8–12 GHz) to several terahertz which opens possibilities for quite new applications of gallium oxide, previously not anticipated.

## 1. Introduction

The materials transparent for a certain electromagnetic bandwidth are key components for many industries such as aeronautic, space, telecommunications, etc. [1,2]. They are called radomes and are configured in the form of various enclosures depending on the applications; their role is to protect antennas from various agents such as rain, snow, dust, heat, etc. The radomes can be seen, for example, in any airport or on top of high buildings. The new wireless communication systems imply that very high frequencies will be used such as 0.1 THz for 5 G and 10 THz for 6 G [3]. There are materials which are transparent in the THz region, such as high resistivity (HR) semiconductors (silicon, boron nitride, gallium arsenide, germanium) or dielectrics (quartz, sapphire, fused silica, diamonds). However, for Internet of Things (IoTs) applications [4], which are the backbone of 5G and 6G communications, very small and lightweight enclosures are required to protect antennas, since the dimensions of antennas are reduced to tens of microns and even lower, comparable to the dimensions of a human hair. Thus, the existing materials for radomes at airports, telecommunications and in many other applications could not be directly reused for tiny IoT due to the requirements of dimensions and weight and there is a real need to find new transparent materials for the applications involved. 

The wide bandgap *β*-Ga_2_O_3_ semiconductor is studied intensively for power electronics [5]. Please note that along with *β*-Ga_2_O_3_, there are other Ga_2_O_3_ polymorphs, all with a small index of refraction (less than 2) [6], indicating that gallium oxide could be a promising candidate for use in transparent electronics. However, little is known about RF properties of Ga_2_O_3_ since only a few results on Ga_2_O_3_ transistors are reported (see [7] and the references therein). Taking into account that three-dimensional architectures consisting of networks of low-dimensional structures prove to be among the multifunctional materials most promising for new applications in electronics and biomedicine [8,9], we developed an ultra-porous architecture made from interconnected microtubes of *β*-Ga_2_O_3_ with nanometer thin walls, carried out its structural characterization and experimentally demonstrated that the new ultra-lightweight nanomaterial, called aero-Ga_2_O_3_ or Aerogallox, is highly transparent and exhibits extremely low reflectivity in the X-band and THz region, up to 3 THz, thus disclosing a novel application of Ga_2_O_3_ previously not anticipated. 

## 2. Materials and Methods 

The technological route for the fabrication of aero-Ga_2_O_3_ is as follows. Initially the aero-GaN was obtained by growing an ultra-thin layer of GaN on sacrificial ZnO templates [10]. The ZnO templates represented networks of interpenetrated ZnO microtetrapods obtained using the flame transport synthesis approach, as previously described in ref. [11]. GaN was grown in a hydride vapor phase epitaxy (HVPE) horizontal reactor containing distinct source and reaction zones. Metallic gallium as well as ammonia (NH_3_, 99.99%, EG No. 231-653.3), hydrogen chloride (HCl, 99.9995%) and hydrogen (H_2_, 99.999%) acquired from Geschaftsbereich Linde Gas, Germany, were used as source materials and carrier gases during the growth process. In the source zone, at high temperature (*T* = 850 °C) the GaCl is formed as a result of chemical reactions between gaseous HCl and liquid Ga. The gaseous GaCl and NH_3_ reacted with each other in the react zone, where initially the temperature was kept at 600 °C for 10 min to initiate nucleation of GaN on the surface of ZnO microtetrapods, and then increased up to *T*_g_ = 850 °C for 10 min to produce GaN layer. In the process of GaN growth, the HCl, NH_3_ and H_2_ flow rates were equal to 15, 600 and 3600 smL/min, respectively. In the process of HVPE growth of GaN, the ZnO sacrificial template is being decomposed due to corrosive atmosphere at high temperature leading to the formation of microtubular structures representing aero-GaN [10]. Previously, we demonstrated the high crystalline quality of the resulting GaN microtubes as well as the existence of ZnO traces (at the level of about 2%) on the inner surface of GaN microtube walls [10,12]. At the final step of the technological route, the aero-GaN is subjected to annealing in air at 950 °C for 60 min and, as a result, is transformed into aero-Ga_2_O_3_ or Aerogallox.

The aero-GaN and aero-Ga_2_O_3_ thin films crystal structure and phases were investigated using a Bruker AXS D8 Advance X-ray diffractometer (XRD, Bruker Italia S.r.l., Milano, Italy) in a standard *θ*–2*θ* Bragg–Brentano configuration with a monochromatic Cu Kα1 (*λ* = 0.15406 Å) radiation. A 40 kV beam voltage and 40 mA beam current were used. For acquisition a linear position-sensitive semiconductor detector (LYNXEYE, Bruker Italia S.r.l., Milan, Italia) in 0D-mode was used, beam optics were Göbel mirror, 6 mm slit, Soller 2.5°; detector optics were 6 mm slit, Soller 2.5°. Diffraction pattern data were collected between 20° and 50° with step lengths of 0.025°, the sample was measured in powder form. 

The chemical composition at surfaces was studied by means of X-ray Photoemission Spectroscopy, XPS (AXIS ULTRA, DLD Kratos Analytical, Manchester, UK) equipped with a monochromatic Al Kα source (h*ν* = 1486.6 eV) operating at 150 W (h—Planck’s constant, *ν*—frequency). Spectra were recorded from a 100 × 100 µm^2^ analysis area and at 160 eV (survey) pass energy, whereas core level spectra were recorded using pass energy of 20 eV. Operating pressure was 6 × 10^−7^ Pa. Prior to the measurement, the surfaces were sputtered using an Ar^+^ beam operated at 2 keV and 0.84 µA for 2 min. Surface charging was compensated using low energy (~5 eV) electrons and adjusted using the charge balance plate on the instrument. Three different spots were analyzed for each sample. All the spectra were processed with CasaXPS (ver. 2.3.20). Spectra were calibrated setting hydrocarbon C1s at 285.0 eV. The surface composition was evaluated from the survey spectra, after a Tougaard U3-type background subtraction, using relative sensitivity factors provided by the manufacturer. Peak fitting was performed with no preliminary smoothing. Symmetric Gaussian–Lorentzian (70% Gaussian and 30% Lorentzian) product functions were used to approximate the line shapes of the fitting components.

**Figure 1:**
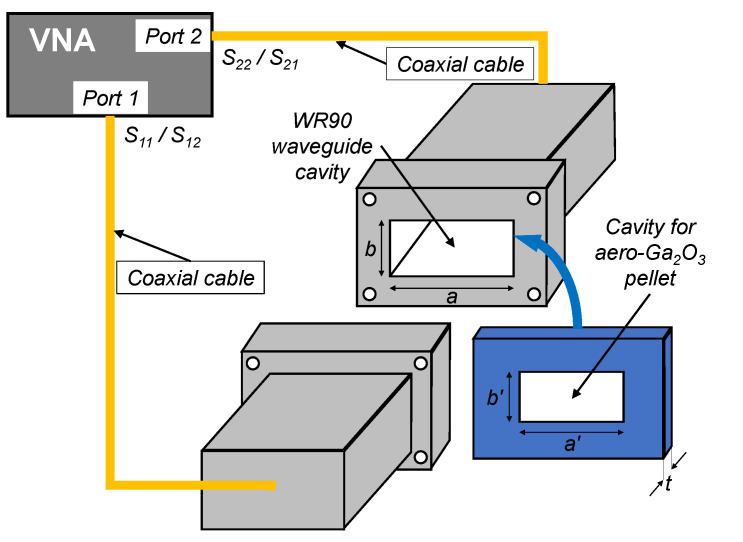
Microwave experimental set-up for X-band characterization of the aero-Ga_2_O_3_.

For the microwaves and terahertz characterizations of the material, bulk samples were prepared in the form of rectangular pellets (20 mm × 10 mm × 2 mm). The freestanding samples were exposed to electromagnetic radiation. The microwave characterization of the aero-Ga_2_O_3_ pellets was carried out by means of a VNA (Vector Network Analyzer) connected to a WR90 waveguide-based set-up suitable for measurements in the X-band (i.e., 8.2–12.4 GHz) [13]. A schematics of the waveguide is presented in Figure 1, where *a* = 22.86 mm and *b* = 10.16 mm. Since the dimensions of the cavity are a bit larger than the aero-Ga_2_O_3_ pellet, we made use of a supporting flange to fix the sample in the cavity, as to avoid electromagnetic radiation due to imperfect coupling between the two waveguides, hence affecting the reliability of the performed measurements.

For terahertz measurements the samples were fixed on metallic holders covering 6 mm aperture, the radiation passing through the sample in free space. Samples with different densities from 70 to 110 mg/cm^3^ of aero-Ga_2_O_3_ were investigated. Room-temperature terahertz spectra of complex dielectric permittivity ε*(*ν*) = *ε*’(*ν*) + i*ε*”(*ν*) were measured in the range *ν* = 10–100 cm^–1^ in a quasi-optical arrangement (in an open space with no waveguides used) with the help of commercial time-domain spectrometer TeraView TPS 3000 (TeraView, Cambridge, UK). The spectra of the real and imaginary parts of dielectric permittivity are determined in the transmission geometry via measurements of the complex transmission coefficient (amplitude and phase) of the plane-parallel samples; standard expressions for electrodynamics of a plane-parallel layer are used [14]. In addition, the spectra of transmission coefficients of the same samples were measured at frequencies up to 7000 cm^−1^ using a standard Fourier-transform spectrometer Bruker Vertex 80v.

## 3. Results and Discussion

### 3.1. Structural Characterization of Aero-Ga_2_O_3_

**Figure 2:**
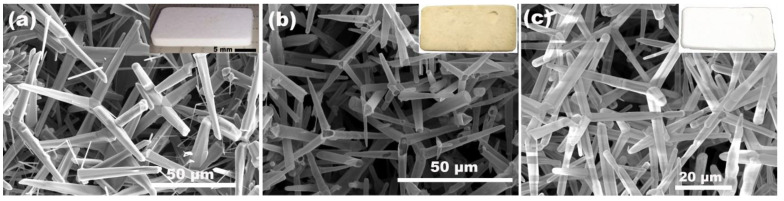
SEM images of (**a**) initial ZnO template, (**b**) intermediate aero-GaN, and (**c**) resulted aero-Ga_2_O_3_ nanomaterial. The inset pictures represent the photographs of the pellet samples of ZnO, GaN, and Ga_2_O_3_ respectively.

Figure 2 depicts scanning electron microscope (SEM) images of interpenetrated networks of ZnO microtetrapods (a), aero-GaN (b) and the obtained aero-Ga_2_O_3_ (c). The inset pictures are the respective photos of the pellets of 20 mm × 10 mm × 2 mm (L × W × H), where one can easily distinguish the change in color of the material after each technological step (white, yellow, white).

**Figure 3:**
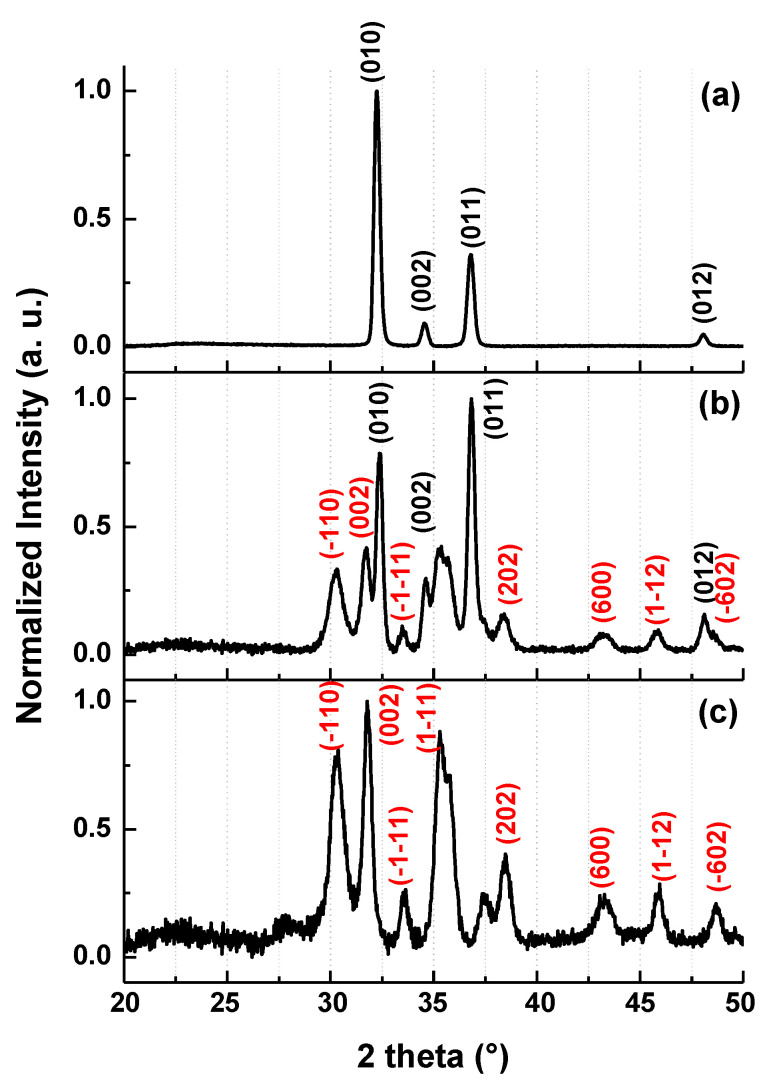
XRD spectra of aero-GaN (**a**), mixture of GaN and Ga_2_O_3_ phases (**b**) and aero-Ga_2_O_3_ (**c**).

Figure 3 illustrates the XRD spectra of aero-GaN (Figure 3a) used for the preparation of aero-Ga_2_O_3_, the mixture phase of GaN and Ga_2_O_3_ (Figure 3b) that was obtained by thermal treatment of aero-GaN at *T* = 800 °C in air for 1 h, and the completely transformed aero-Ga_2_O_3_ (Figure 3c) obtained after 1 h of treatment at 950°C of aero-GaN samples. The XRD reflections at 32.25°, 36.8° and 48.12° were assigned to wurtzite GaN planes (010), (011) and (012), respectively, while the reflections at 30.3°, 31.7°, 33.5°, 35.3°, 38.4°, 43.2°, 45.9° and 48.9° were assigned to beta phase of monoclinic Ga_2_O_3_ planes (−110), (002), (−1−11), (1−11), (202), (600), (1−12) and (−510), respectively. Comparing the different patterns, the qualitative trend of GaN oxidative phase change occurring at different stages of the thermal synthesis processes can be appreciated. While both nitride and oxide phases co-exist after the thermal step at *T* = 800°C in air for 1 h (both reflection peaks sets for wurtzite GaN and monoclinic Ga_2_O_3_ are found in the diffraction pattern), only reflections from the oxide remain after annealing at *T* = 950°C. Formation of monoclinic Ga_2_O_3_ in thin structures was already observed for annealing temperatures as low as 750 °C [15].

**Figure 4:**
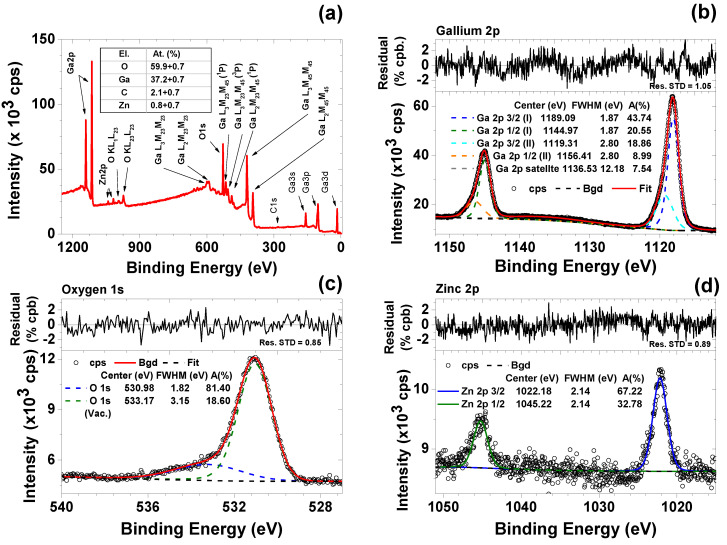
(**a**) XPS survey spectra of *β*-Ga_2_O_3_ powder with attribution of the principal emission lines, table in the inset shows the derived atomic abundancies. (**b**–**d**) High resolution XSP spectra with resolved peak components and normalized residuals, of the Ga 2p (**b**), O 1s (**c**) and Zn 2p (**d**) regions.

Figure 4 shows the principal results of *β*-Ga_2_O_3_ surface chemical analysis by means of XPS. Survey spectra (Figure 4a) show that the main elements left in the sample after annealing are Ga and O with low level of C and Zn present as impurities. Observed gallium main emission lines are Ga 2p (shown in Figure 4b), Ga 3p and Ga 3d (not shown) and several Auger lines in the 400–600 eV regions. All Ga doublets show energy shifts with respect to metallic gallium binding energy (BE), consistent with literature reported values for *β*-Ga_2_O_3_ [16,17]. Data show that O 1s peak (Figure 4c) need to be resolved using two components. The main component BE at 530.98 eV can be attributed to Ga-O bonding in the oxide while the higher BE contribution at 533.17 eV can be assigned to O-vacancies sites and/or Ga suboxides [18,19,20]. This peak presence is related to the Ar ions sputtering process used to clean the sample surface in order to remove adventitious carbon contamination. Presence of reduced Ga can be seen also in the low energy satellite peak (at BE 19.16 eV) of Ga 3d (not shown). However, sputter cleaning of the sample was necessary for reliable estimation of atomic concentrations; before Ar^+^ bombardment C atoms surface concentration was estimated to be around 11% while after cleaning dropped down to 2%. Measured Ga/O ratio is thus 0.62 with an estimated relative error of 6.21% (stoichiometric value 0.67). The presence of Zn 2p doublet (Figure 4d) suggests some residual (<1 at. %) of metallic impurities left from the sacrificial ZnO matrix used in the fabrication process. It is to be noted that the chemical composition study using energy dispersive X-ray analysis disclosed the stoichiometric composition of the Ga_2_O_3_ nanoarchitecture, at the same time traces of Zinc at the level of about 1.5 at. % were shown. 

### 3.2. Characterization of Aero-Ga_2_O_3_ at Microwaves.

**Figure 5:**
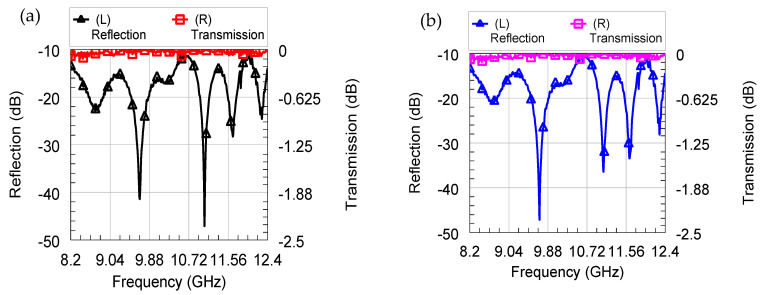
Microwave measurements in X-band for the two cases: (**a**) without the aero-Ga_2_O_3_ pellet and (**b**) with the aero-Ga_2_O_3_ pellet, in terms of reflection (left vertical axis, solid black and blue curves) and transmission (right vertical axis, solid red and pink curves).

The S-parameters at the two ports of the VNA were measured to provide the reflection (S_11_/S_22_) and transmission (S_12_/S_21_) coefficients at/between the two ports, respectively. The measured S-parameters of the Aerogallox sample (Figure 2c) with the density of 110 mg/cm^3^ are depicted in Figure 5.

In Figure 5a,b we show only S_11_ and S_21_, since S_22_ is identical with S_11_, and S_12_ with S_21_ thanks to reciprocity and symmetry of the scattering matrix (typical for passive components). One can notice that the reflection coefficient is better than −10 dB all over the band of interest, whereas the insertion loss has a maximum value better than −0.12 dB in both cases without and with aero-Ga_2_O_3_. In other words, the presence of the aero-Ga_2_O_3_ inside the cavity between the two X-band waveguides does not affect at all the transmission of the microwave signal, which means the aero-Ga_2_O_3_ is completely (within our accuracy) electromagnetically transparent in the X-band. 

### 3.3. Characterization of Aero-Ga_2_O_3_ in the Terahertz Region

**Figure 6:**
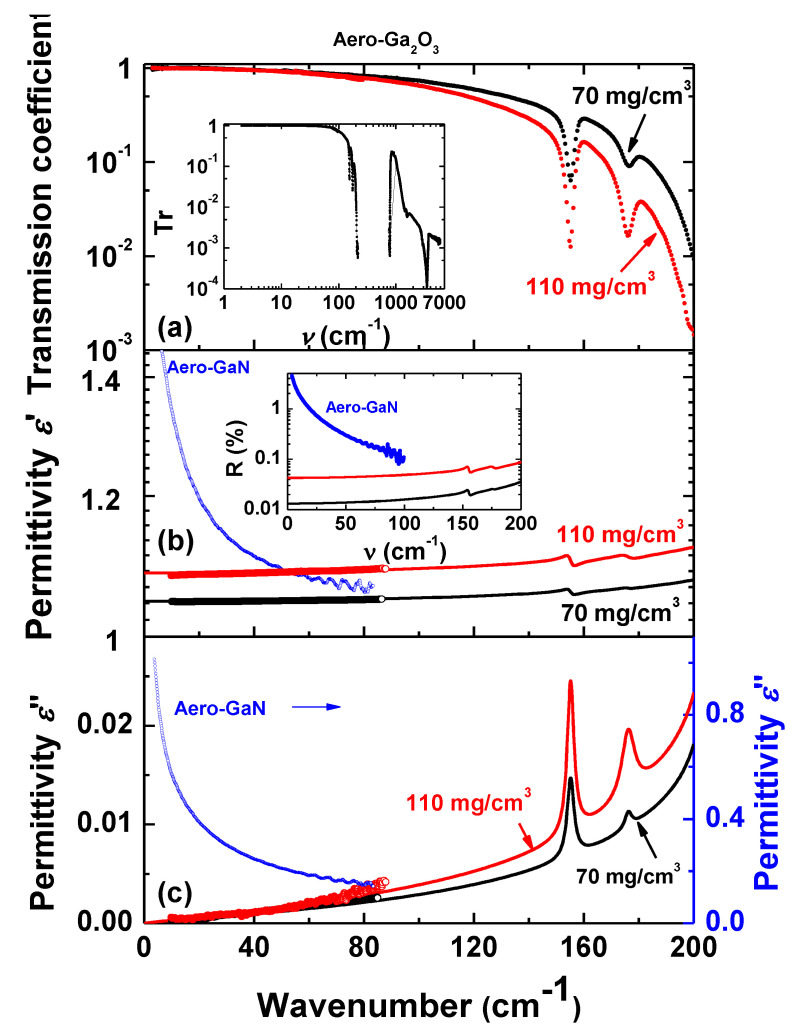
Terahertz spectral characteristics of aero-Ga_2_O_3_ samples with two different densities of 70 mg/cm^3^ and 110 mg/cm^3^, and aero-GaN sample with the density of 15 mg/cm^3^ (data from [22]): transmission coefficient *Tr* (**a**), real *ε*′ (**b**) and imaginary *ε*” (**c**) parts of dielectric permittivity. Open dots on panels (**b**) and (**c**) present THz data for permittivity. Inset in panel (**a**): spectrum of transmission coefficient measured at frequencies up to 7000 cm^−1^. Inset in panel (**b**): spectra of reflection coefficient calculated basing on measured spectra of real and imaginary parts of dielectric permittivity using standard Fresnel expressions [14].

Two samples of Aerogallox with different densities were studied—the low density equals to 70 mg/cm^3^ and the high density was 110 mg/cm^3^. Comparative analyses with aero-GaN samples with the density of 15 mg/cm^3^ were performed. Broad-band terahertz-infrared transmissivity spectra for two samples with low and high densities are shown in Figure 6a. As expected, more dense samples have lower transmissivity. A decrease in the transmission coefficient for frequencies growing up to 200 cm^−1^ is caused by intensive absorption between 200 and 800 cm^−1^ (see inset in Figure 6a). Our attempts to extract information about absorption mechanisms in this range by measuring the spectra of reflection coefficient failed due to very low intensity reflected by the samples, i.e., extremely small value of reflectivity, as discussed below. Below 200 cm^−1^, there are two narrow absorption resonances located at ~155 and ~176 cm^−1^ whose parameters (frequency position and intensity) do not depend noticeably on the sample density. A few more resonances are observed above 800 cm^−1^, most intensive at ~3500 cm^−1^. Origin of observed narrow absorptions seems to be related to the presence of impurities such as hydrogen [21].

**Figure 7:**
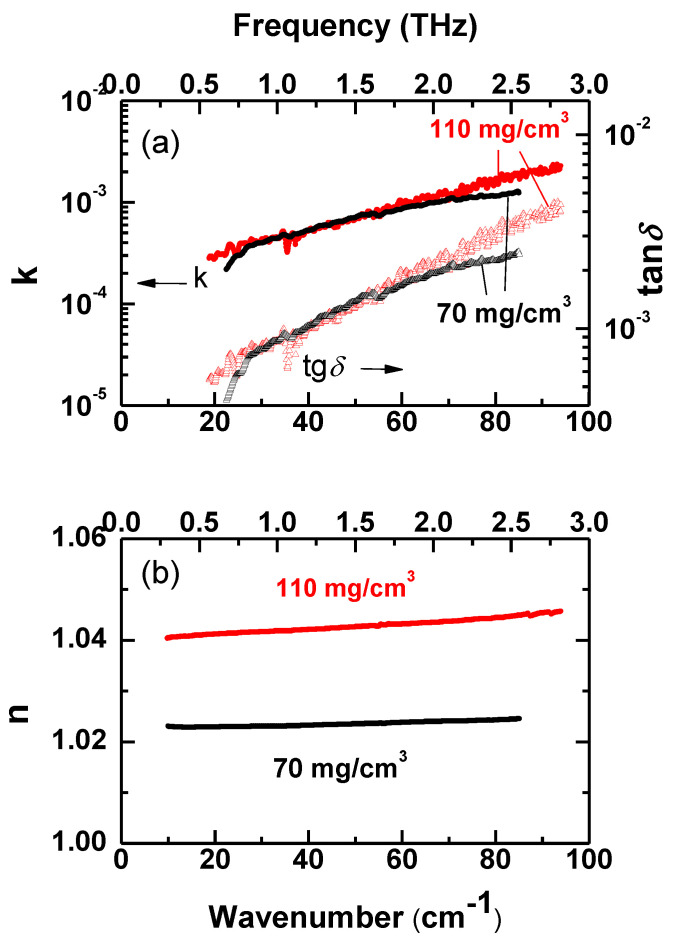
Spectra of terahertz electrodynamic characteristics of aero-Ga_2_O_3_ samples with two different densities of 70 mg/cm^3^ and 110 mg/cm^3^: imaginary *k* (**a**) and real *n* (**b**) parts of complex refractive index *n* + i*k* and dielectric loss tangent tan*δ* (panel a, triangles).

In Figure 6b,c we present the spectra of real and imaginary permittivity of the two samples, together with the reflection coefficient. It is seen that THz characteristics of our aero-Ga_2_O_3_ material drastically differ from those of parent bulk Ga_2_O_3_ crystal as well as from any other bulk semiconductor. For example, typical semiconductors Ge, GaAs and Si have refractive indexes at 300 GHz *n* = 3.99, 3.59 and 3.43, respectively [23], while the refractive index of aero-Ga_2_O_3_, *n* ≈ 1.07 (sample with density 70 mg/cm^3^, Figure 7), only slightly exceeds that of vacuum (*n* = 1). From Figure 6b,c one can see that there is pronounced difference between terahertz response of aero-Ga_2_O_3_ and previously studied aero-GaN [22]. While in aero-Ga_2_O_3_ real permittivity *ε*′ is dispersionless and imaginary permittivity *ε*” is small and approaches zero with frequency decrease, both indicating absence of any absorption process, aero-GaN demonstrates strong increase of *ε*′ and *ε*” toward low frequencies. The origin of corresponding pronounced absorption is associated with the polarizability of the 3D architecture of mutually interpenetrated GaN aerotetrapods, with the ZnO-GaN interfaces, and finally with dynamics of relatively big complexes of tetrapods. No such contribution exists in the present Aerogallox nanomaterial. Aero-Ga_2_O_3_ is characterized by very small imaginary permittivity *ε*” and dielectric losses tan*δ = ε*”/*ε*′ (Figure 7a) that are responsible for radiation absorption, and by real part of permittivity *ε*′ and real part *n* of complex refractive index n* = *n* + i*k* (Figure 7b) close to 1. Both factors lead to a very low reflection coefficient (inset in Figure 6b) $R=\left[{\left(n-1\right)}^{2}+k^{2}\right]/\left[{\left(n+1\right)}^{2}+k^{2}\right]\approx \left[{\left(\sqrt{\varepsilon ’}-1\right)}^{2}+k^{2}\right]/\left[{\left(\sqrt{\varepsilon ´}+1\right)}^{2}+k^{2}\right]\;$that is as small as *R* ≈ 0.1% and can be made close to *R* ≈ 0.01%. We can compare these extremely low reflectivity values with those of typical bulk semiconductors, mentioned above, Ge (*n* = 3.99), GaAs (*n* = 3.59) or silicon (*n* = 3.43); corresponding reflection coefficients fall in the range 30–36%. Along with high value of transmissivity, this low reflectivity of the material may be beneficial for future applications of Ga_2_O_3_.

## 4. Conclusions

We developed a highly porous ultra-lightweight three-dimensional nanoarchitecture consisting of interconnected microtubes of Ga_2_O_3_ with nanometer thick walls, and demonstrated that the gallium oxide skeleton is of crystalline β-phase with almost stoichiometric composition. The new nanomaterial is shown to exhibit ultra-low reflectivity and high transparency in an extremely wide range of the electromagnetic spectrum, covering the X-band and THz region, up to 3 THz. The disclosed novel properties of aero-Ga_2_O_3_ open possibilities, in premiere, for the use of gallium oxide in IoT applications.
